# Chengru Feng: The founder of biological illustration in China

**DOI:** 10.1007/s13238-016-0249-6

**Published:** 2016-02-13

**Authors:** Hong Jiang

**Affiliations:** Center for Culture, Science and Technology, Sichuan University, Chengdu, 610065 China

The eighteenth and nineteenth century was the heyday of biological illustration in western world. However, China did not have the tradition of scientific biology as early as the West, though it had a long history of herbalism. Biology was not introduced into this country until the late nineteenth century, around the end of Qing Dynasty, while the practice of biological illustration was even much later than that. In ancient China, a lot of illustrated books did exist, especially herbals, which also contained animal illustrations, since traditional Chinese medicine often used both plant and animal materials. However, it is hard to say whether these illustrations were scientifically accurate and correct, because they were drawn mainly according to experience, not scientific standard. As the founder of biological illustration in China, Chengru Feng (1896–1968), not only drew for botanists and zoologists, such as Xiansu Hu, Huanyong Chen, and Bing Zhi, but also trained the earliest Chinese biological illustrators, who greatly contributed to the progress of biology in China. He also published *Biological Illustration*, which was the first Chinese handbook in this field.

**Figure 1:**
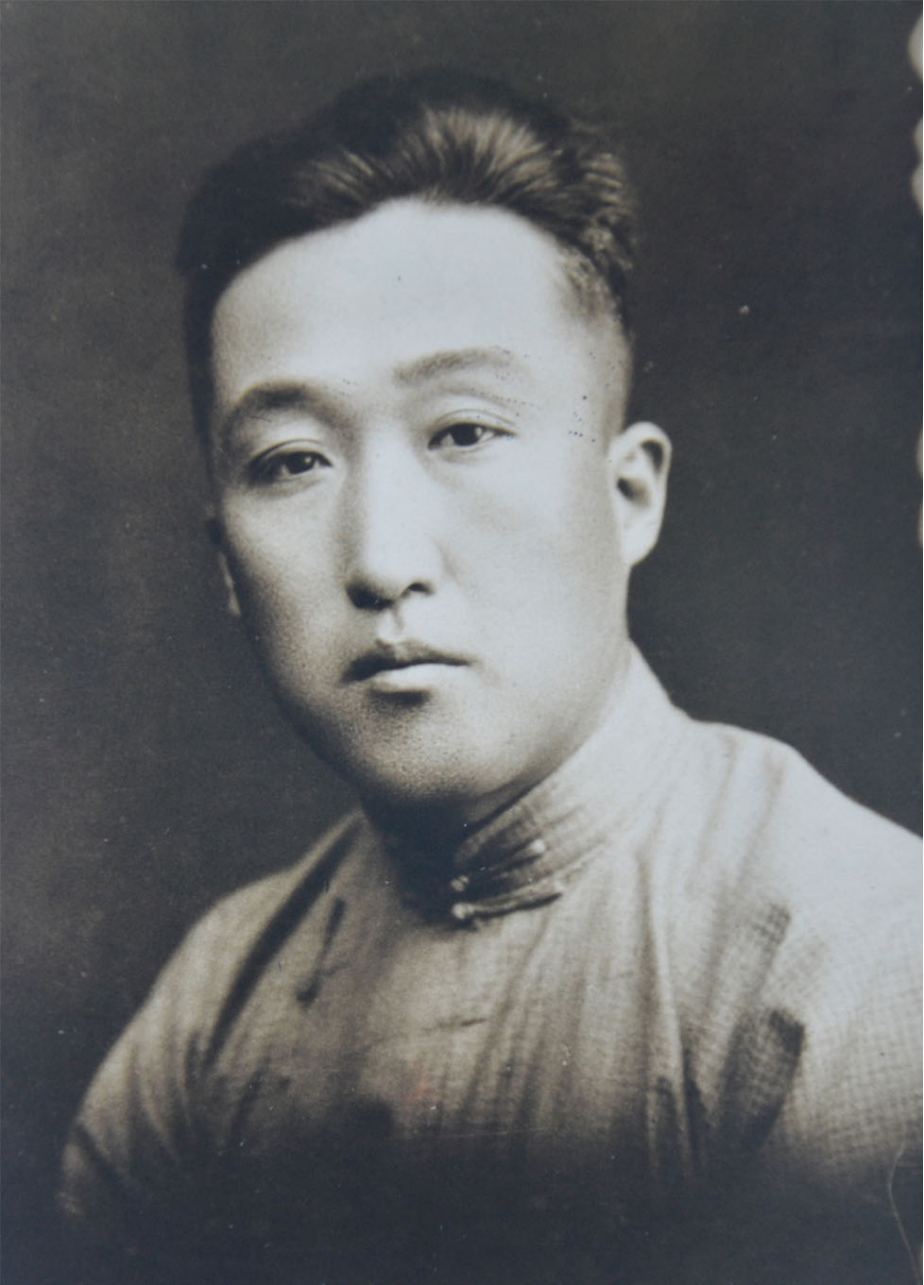
Chengru Feng (1896–1968)

**Figure 2:**
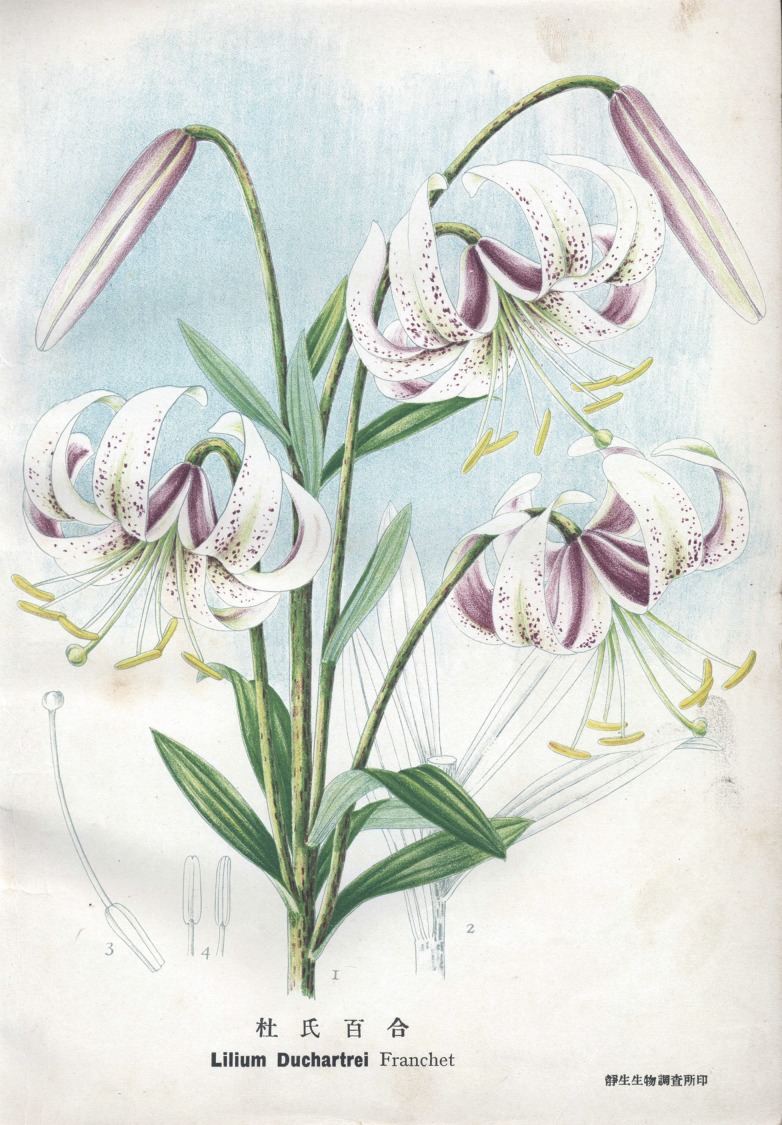
Illustration of *Lilium Duchartrei* painted by Chengru Feng

Affected by his family, Chengru Feng, whose father ran an old-style private Chinese school and was also engaged in farming, entered a normal college and then became an art teacher in an elementary school at the age of twenty. As an artist, Chengru Feng was gifted at traditional Chinese painting, pencil sketch, still life, and also western oil painting and water colors as well. His biological illustrations combined techniques of Chinese claborate-style painting and western painting, whilst exhibiting his own style. From 1921 to 1922, when teaching art in National Nanjing Higher Normal School (now Nanjing Normal University), Chengru Feng was invited by botanist Xiansu Hu and zoologist Bing Zhi to draw dozens of large biological illustrations for a newly established biological department in Southeast University and Biological Institute of Chinese Scientific Society. This was the first time that Chengru Feng drew for science and cooperated with early top biologists in China. After this experience, he began to draw for botanical and zoological publications and worked with more biologists. His first published set of biological illustrations appeared in Zhen Chen’s paper on the genetics of gold fishes, which was considered as the beginning of biological illustration in China Sun et al. 2010.

Most of Chengru Feng’s illustrations were botanical ones. In 1925, he drew all illustrations for Huanyong Chen’s book *Shu Mu Tu Shuo* (*Illustrated Book of Trees*), which was the first illustrated modern-botany book drawn by a Chinese illustrator. During the next ten years, he had drawn 250 botanical illustrations for Vol. 1–Vol. 5 of *Icones Plantarum Sinicarum*, edited by Xiansu Hu and Huanyong Chen. Later, he drew over 200 illustrations for Renchang Qin’s *Icones Filicum Sinicarum* Botanical Society of China 1994. These illustrated books opened a new era of botany in China.

Besides drawing illustrations, Chengru Feng trained dozens of biological illustrators. He first trained a few of his family members to illustrate, including his younger brother Zhanru Feng, son Zhongyuan Feng, nephews Xingsheng Jiang, Xingqiang Jiang, and Weicheng Zong, etc. These people were then later engaged in biological illustration in academic institutes of biology around the country. His family soon became well known by their illustrations. The title “Flowers of Feng family” was given to this family Zhou 1981, to praise their great botanical illustrations. Chengru Feng continued to train more people in specialized art classes and in 1943, supported by Xiansu Hu and Bing Zhi, he established “Specialized Class of Biological Illustration, Jiangnan Art College” in his hometown. There Chengru Feng taught art related courses, including art history, skills of biological illustration, theory of painting, calligraphy, and so on. He also invited Jin Tang, a professor of Jingsheng Institute of Biology, to teach English and botany and subsequently invited entomologist Zongbao Zhang to teach zoological taxonomy Sun et al. 2010. Over 20 biological illustrators graduated from Chengru Feng’s class and became engaged in drawing illustrations for marine biology, herbal, medical science, botany, zoology, etc. Some of them became renowned as the best biological illustrators in China. During the Anti-Japanese War and War of Liberation, only a few biological illustrators continued to work in China and the practice of biological illustration almost disappeared. Therefore, Chengru Feng’s efforts following the war played an extremely significant role in the revival of biological illustration in modern China.

In 1959, Chengru Feng’s book *Biological Illustration* was finally published, though he began to summarize his knowledge about biological illustration as early as when he trained his family members to draw in 1929. When teaching in the specialized class, he edited this book systematically for his students, but never published it. It was comprehensively improved during the spring of 1955, when he was invited to give lectures by Nanjing Agricultural College (Feng 1959). This handbook provided a comprehensive introduction to biological illustration, including: requirements of biological illustration; tools, materials, instruments needed and their operation; basic skills and different ways of drawing; how to draw animals (insect and fish) and plants (flowering plants and algae); technology of printing, and so on. Chengru Feng especially emphasized that a biological illustration needed to be scientifically correct. No matter the shape, ratio, color or other details of an illustration should be completely correct and accurate (Feng 1959). He was very conscious of differences and connections between biological illustration and painting. Since most illustrations were drawn for printing, rather than appreciation as paintings, he also improved the printing techniques of illustrations. As the first handbook of biological illustration, *Biological Illustration* revealed the efforts and contributions Chengru Feng had made for this field. It also served as the benchmark text book for later illustrators.

Besides his achievements mentioned above, Chengru Feng also helped Xiansu Hu with the establishment of Jingsheng Institute of Biology. In 1928, he moved to Beijing with Xiansu Hu, who devoted himself to establishing this new scientific institute. Chengru Feng later became a researcher of botanical department, an illustrator, and also the director of the printing house of Jingsheng Institute. In 1929, he attended the Fourth Pacific Science Conference with Xiansu Hu in Indonesia. During that conference, he drew a lot of illustrations from Chinese plants specimens collected by western countries, which was helpful for the publication of Vol. 3 and Vol. 4 of *Icones Plantarum Sinicarum* Sun et al. 2010.

Marginalized in the scientific community, biological illustrators face a difficult future in present day China. Consequently, there are fewer and fewer illustrators working in academic institutes. There are a few reasons for this: the rapid progress of photography, the evaluation system of science research, the shift from traditional taxonomy to microbiology, etc. However, when we look back the development of biology in the past century in China, it is easy to see how important biological illustrations have been for this field. Therefore we should not forget early biological illustrators, especially the founder of biological illustration in China: Chengru Feng.
